# Visual outcome, safety, and complications of iris suture fixation versus iris claw fixation for aphakic correction: a prospective randomized study

**DOI:** 10.1186/s12886-026-04785-x

**Published:** 2026-04-14

**Authors:** Ehab Mohamed Elsayed Saad, Hazem Elbadry Mohammed Mohammed, Ehab Tharwat, Ahmed Mohammed Sakr, Sayed Mostafa Elsayed Abdelhafeez, Nehad Mohammed Yusef, Mohamed Abdulbadiea Abdulgyed Rashed, Ahmed Mohamed Raafat Tawfik, Mohamed Alsadawy Hassan Alsadawy, Amro Radi Mahmoud Abdou, Mohamed Gaber Okasha, Mohamed Saad Ibrahim Alabshihy, Ahmed Samy Abdelmonem Hussein, Ahmed Mohamed Ahmed Ghazala, Ghazy Basiony Hassan Abd Elkader, Mohamed abdel gaffar Mohamed elhaw, Mohamed Abd Elmoamen Mohamed Saad Eldeen, Mahmoud Abdelhalim Ali Ali, Sherif Salah Eid Elsayed, Tamer Gamal El Sayed

**Affiliations:** 1 Department of Ophthalmology, Faculty of Medicine, Benha University, Benha, Egypt; 2 Department of Ophthalmology, Faculty of Medicine, Al-Azhar University, Cairo, Egypt; 3 Department of Ophthalmology, Faculty of Medicine, Al-Azhar University, Damietta, Egypt; 4 Department of Ophthalmology, Faculty of Medicine, Aswan University, Aswan, Egypt; 5 Department of Ophthalmology, Faculty of Medicine, Fayoum University, Al- Fayoum, Egypt; 6 Department of Ophthalmology, Faculty of Medicine, Zagazig University, Zagazig, Egypt

**Keywords:** Aphakia, Iris-claw lens, Iris-suture fixation, Posterior chamber intraocular lens, Secondary IOL

## Abstract

**Background:**

This study aimed to compare visual outcomes, safety profiles, and complication rates between retropupillary iris claw fixation and iris suture fixation of posterior chamber intraocular lenses (IOLs) in aphakic eyes lacking sufficient capsular support.

**Methods:**

This prospective randomized controlled trial enrolled 50 aphakic eyes from 50 patients, equally allocated to iris claw fixation (*n* = 25) or iris suture fixation (*n* = 25). The primary outcome was corrected distance visual acuity (CDVA) at 12 months. Secondary outcomes included surgical duration, IOL decentration and tilt, intraocular pressure (IOP), cystoid macular edema (CME), endothelial cell density loss, and other postoperative complications. Evaluations were performed at 1, 3, 6, and 12 months postoperatively.

**Results:**

Both techniques yielded statistically significant CDVA improvement from baseline (*p* < 0.001). Mean surgical duration was significantly shorter in the iris-claw group (30.5 ± 3.2 min) compared with the iris-suture group (46.4 ± 4.5 min; *p* < 0.001) reflecting the greater technical complexity of suture-based fixation. Regarding cost, no formal economic analysis was performed; however, while iris-claw lenses are specialized and iris-suture fixation requires 10 − 0 Prolene, both techniques use readily available surgical materials, and the overall difference in procedural cost is expected to be modest. The iris claw group demonstrated superior early visual recovery at 1 and 3 months; however, CDVA converged between groups by 12 months (0.10 ± 0.10 vs. 0.12 ± 0.10 logMAR; *p* = 0.23). Refractive outcomes modestly favored iris claw fixation, with lower mean spherical equivalent (− 0.30 ± 0.40 diopters vs. −0.50 ± 0.50 diopters; *p* = 0.08) and reduced residual astigmatism (− 0.85 ± 0.61 diopters vs. −1.21 ± 0.74 diopters; *p* = 0.04). IOP, central corneal thickness, and macular thickness remained stable throughout follow-up in both cohorts. Complication rates were low: disenclavation occurred in one eye (4%) in the iris-claw group, mild suture erosion in two eyes (8%) in the iris-suture group, and transient anterior chamber inflammation in three eyes (12%) and four eyes (16%), respectively. Endothelial cell loss was significantly greater in the iris claw group (14.5% vs. 9.8%; *p* < 0.001).

**Conclusions:**

Retropupillary iris claw and iris suture fixation both provide effective and safe options for secondary IOL implantation in aphakic eyes without capsular support. Iris claw fixation facilitates faster early visual rehabilitation and marginally better refractive predictability, whereas iris suture fixation may be preferable in eyes with preexisting endothelial compromise. Surgical technique selection should be individualized based on ocular comorbidities and the surgeon’s expertise.

**Trial registration:**

Retrospectively registered at ClinicalTrials.gov; registration number: NCT06933654; registered on 11 April 2025.

**Supplementary Information:**

The online version contains supplementary material available at 10.1186/s12886-026-04785-x.

## Background

Aphakia, the absence of the crystalline lens, typically arises from complicated cataract surgery, ocular trauma, or congenital anomalies [1, 2]. Visual rehabilitation in aphakic eyes lacking adequate capsular support presents a surgical challenge, as the absence of the capsular–zonular complex precludes standard in-the-bag intraocular lens (IOL) implantation [3, 4]. Consequently, alternative fixation strategies have been developed, including anterior chamber angle-supported IOLs, anterior chamber iris-fixated IOLs, posterior chamber iris-fixated IOLs, and scleral-fixated posterior chamber IOLs [5, 6].

Although anterior chamber IOLs offer relatively straightforward implantation, their long-term use is associated with complications such as secondary glaucoma, chronic uveitis, hyphema, progressive corneal endothelial cell loss, and bullous keratopathy. These adverse outcomes stem largely from angle crowding, altered aqueous humor dynamics, and chronic endothelial proximity or trauma [7, 8]. For these reasons, posterior chamber IOL implantation is generally preferred, as it better restores normal ocular anatomy, enhances optical stability, and reduces the risks of pseudophacodonesis and endothelial compromise [9].

Among posterior chamber techniques, iris suture fixation and retropupillary iris-claw fixation have gained increasing attention [10]. Iris suture fixation secures a posterior chamber IOL to the mid-peripheral iris using sutures, positioning the optic behind the iris plane [11, 12]. This approach permits the use of foldable IOLs through smaller incisions and preserves anterior chamber anatomy. However, it is technically demanding and may lead to suture degradation, IOL tilt or decentration, chronic low-grade inflammation, elevated intraocular pressure (IOP), and cystoid macular edema (CME) [13]. These complications are mechanistically related to suture–iris interaction, prolonged intraocular manipulation, and potential delayed suture failure.

Retropupillary iris-claw IOLs, such as the Artisan aphakia lens, feature a vaulted, sutureless design that enclavates to the posterior surface of the mid-peripheral iris, positioning the optic within the posterior chamber [14]. By eliminating sutures and maintaining a retropupillary location, this technique aims to minimize endothelial cell loss, reduce chronic inflammatory stimuli, and preserve physiologic aqueous humor flow [15]. Clinical studies report satisfactory visual outcomes and acceptable complication rates with this approach; however, concerns persist regarding disenclavation, localized iris trauma, pupil distortion, and potential effects on long-term iris integrity [16, 17].

Despite the growing use of both techniques, high-quality prospective comparative data remain limited. Existing studies are often retrospective, involve small cohorts, or employ heterogeneous outcome definitions and follow-up intervals, hindering meaningful comparisons of visual recovery, refractive stability, and safety profiles. Furthermore, differences in surgical anatomy, particularly with respect to iris interaction, fixation mechanics, and operative duration, may differentially affect postoperative inflammation, IOP dynamics, IOL centration, and macular status. These considerations underscore the need for a structured comparative evaluation.

Accordingly, this study was designed as a prospective randomized controlled trial to compare retropupillary iris-claw fixation and iris suture fixation of posterior chamber IOLs in aphakic eyes without sufficient capsular support. The primary objective was to evaluate postoperative visual performance using best-corrected visual acuity (BCVA). Secondary outcomes included measures of optical stability and safety: IOL centration and tilt, postoperative IOP behavior, and the incidence of surgery-related complications such as CME.

## Methods

### Study design and participants

**Fig. 1:**
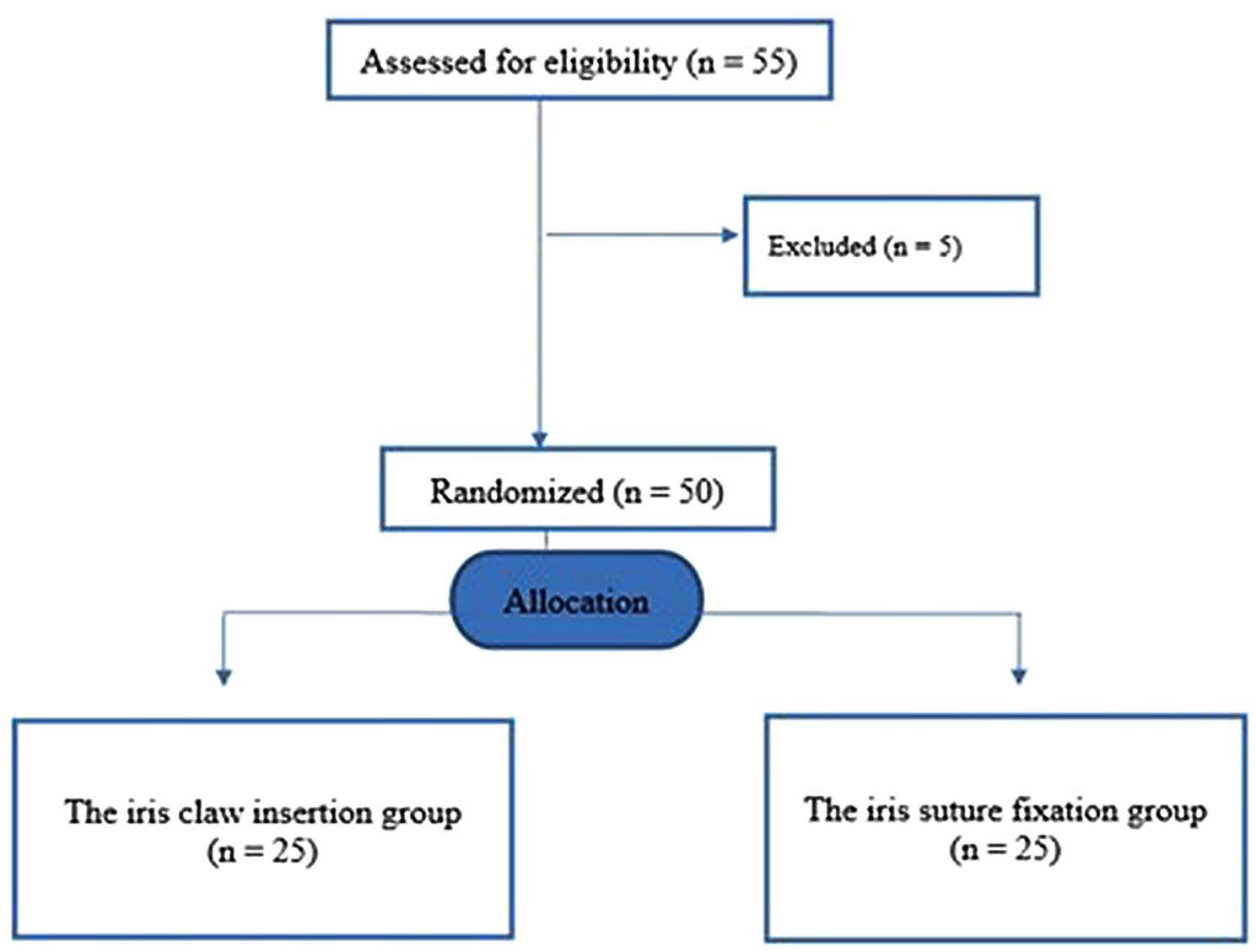
Study flowchart. CONSORT flow diagram illustrating participant enrollment, randomization, allocation, and follow-up in the study

This prospective, randomized controlled trial was conducted at Al-Azhar University Hospitals and Benha University Hospitals between January 2024 and July 2025. The study protocol adhered to the tenets of the Declaration of Helsinki and received approval from the Institutional Review Board of Al-Azhar University Hospitals and Benha University Hospitals (IRB No.: Ophth_28/2024). Written informed consent was obtained from all participants prior to enrollment. Retrospectively registered at ClinicalTrials.gov; registration number: NCT06933654; registered on 11 April 2025. This randomized controlled trial was conducted and reported in accordance with the Consolidated Standards of Reporting Trials (CONSORT) 2025 guidelines. Fifty aphakic eyes from 50 patients with insufficient capsular support were enrolled. Participants were randomly assigned in a 1:1 ratio to undergo either retropupillary iris-claw IOL fixation or iris-suture fixation, using a computer-generated randomization sequence (Fig. 1). Allocation concealment was maintained with sequentially numbered, opaque, sealed envelopes opened immediately before surgery. Given the inherent differences between the surgical techniques, masking of the operating surgeons was not feasible. Postoperative outcome assessors were not involved in the surgical procedures and performed standardized examinations independent of the operating surgeons; however, complete masking could not be guaranteed, as differences in incision length and the presence of sutures might reveal the surgical technique, potentially introducing detection bias. The trial protocol is available from the corresponding author upon reasonable request. No changes to the protocol were implemented after trial commencement. Patients were not involved in trial design or conduct, given the surgical nature of the interventions. Randomization sequence generation was performed by an independent statistician; participant enrollment and allocation envelope opening were conducted by a research coordinator not involved in outcome assessment. All 50 randomized participants completed a 12-month follow-up with no missing data for primary or secondary outcomes. No prespecified subgroup or sensitivity analyses were planned.

Inclusion criteria included Aphakia with CDVA of 4/60 or better, resulting from complicated cataract surgery, ocular trauma, or dislocated or subluxated IOLs. Meanwhile, exclusion criteria included corneal decompensation or endothelial cell density < 1500 cells/mm², posterior segment pathology limiting visual potential (e.g., CME, choroidal neovascularization), insufficient or abnormal iris tissue (including iris neovascularization), and uncontrolled or refractory glaucoma.

### Surgical techniques/interventions

All procedures were performed by experienced anterior segment surgeons following standardized protocols. Anterior vitrectomy was performed when vitreous prolapse into the anterior chamber was present, ensuring complete removal of vitreous strands prior to IOL implantation.

#### Retropupillary iris-claw fixation

**Fig. 2:**
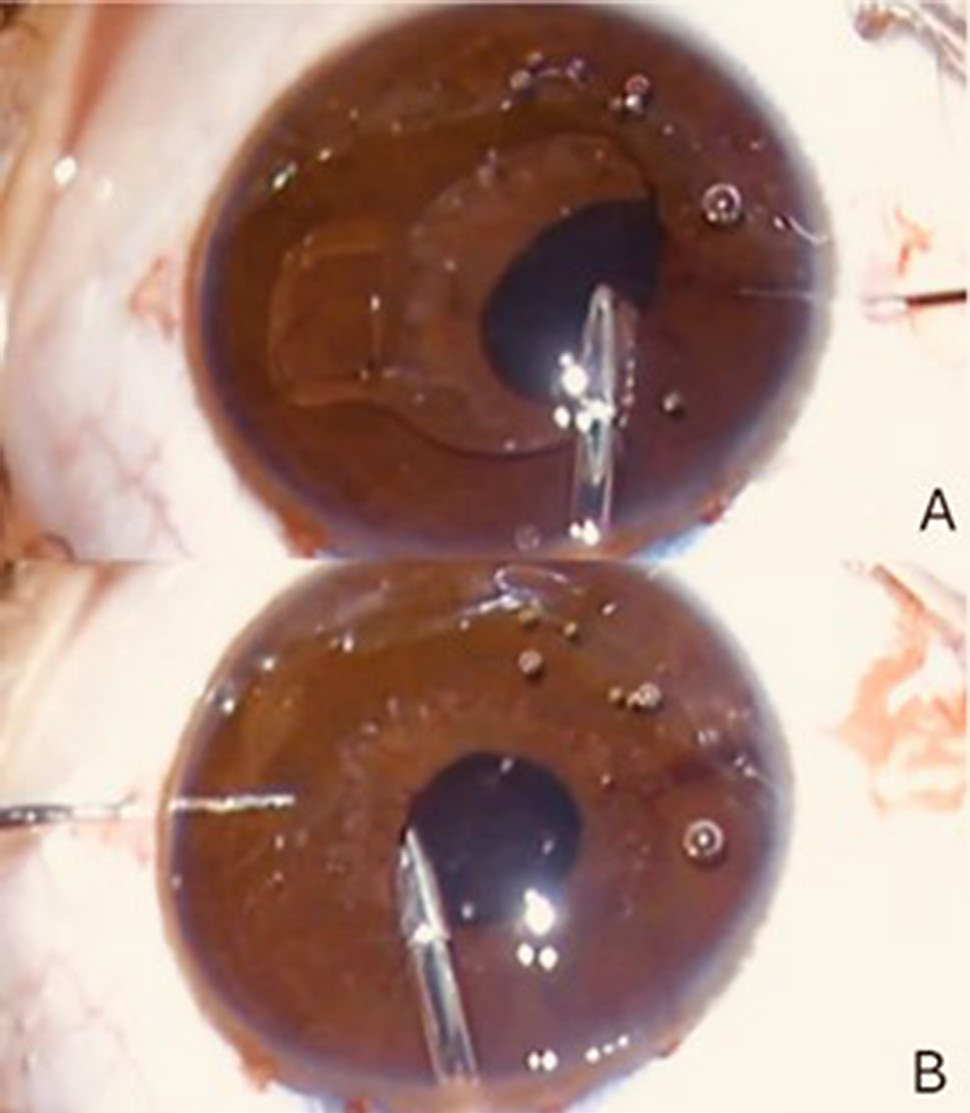
Intraoperative view of enclavation during retropupillary iris-claw intraocular lens (IOL) implantation. The claw haptic captures the mid-peripheral posterior iris stroma, confirming correct retropupillary positioning of the optic posterior to the iris plane. This placement aims to minimize corneal endothelial contact and preserve physiologic aqueous humor dynamics

The Artisan aphakia IOL *(Ophtec BV*,* Groningen*,* The Netherlands)* was implanted using a retropupillary approach through a 5.5–6.0 mm superior corneal incision, with two side-port paracenteses. After deepening the anterior chamber and inducing pharmacologic miosis, the IOL was introduced posterior to the iris plane. Enclavation of the mid-peripheral posterior iris stroma into the haptic claws was performed using a dedicated enclavation needle introduced through paracentesis incisions. A two 10 − 0 nylon sutures were used for wound closure. A peripheral iridotomy was performed when deemed necessary to prevent pupillary block. The main incision was closed using interrupted 10 − 0 nylon sutures. Symmetric enclavation and optic centration were ensured to minimize tilt, reduce endothelial contact, and preserve physiologic aqueous humor dynamics **(**Fig. 2**).**

#### Iris-suture fixation

**Fig. 3:**
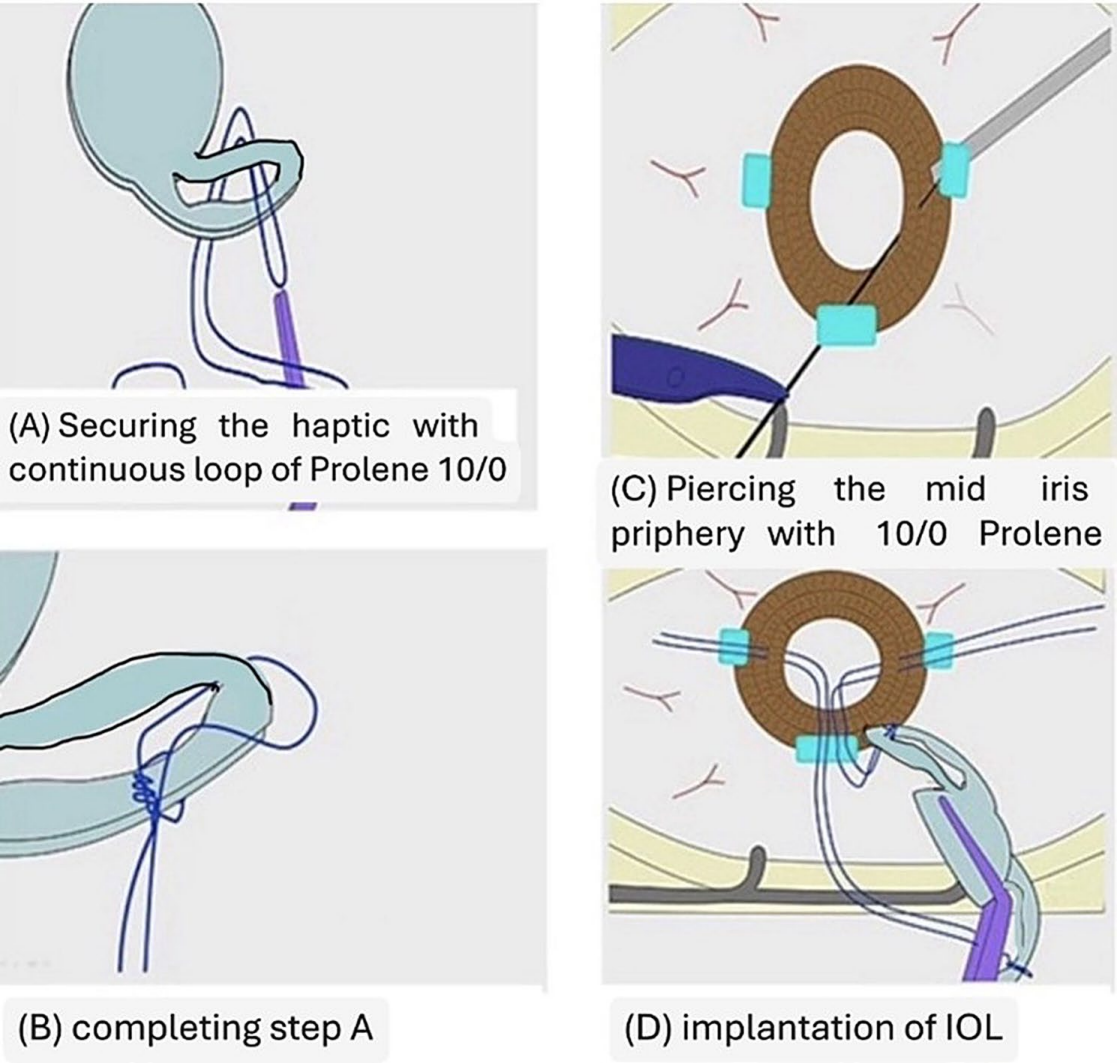
Surgical steps for iris-suture fixation of a foldable posterior chamber intraocular lens (IOL). **(A)** A continuous loop of 10 − 0 polypropylene suture secures the IOL haptic. **(B)** The suture loop is completed around the haptic. **(C)** The suture needle pierces the mid-peripheral iris. **(D)** Final implantation with the IOL secured to the iris stroma

**Fig. 4:**
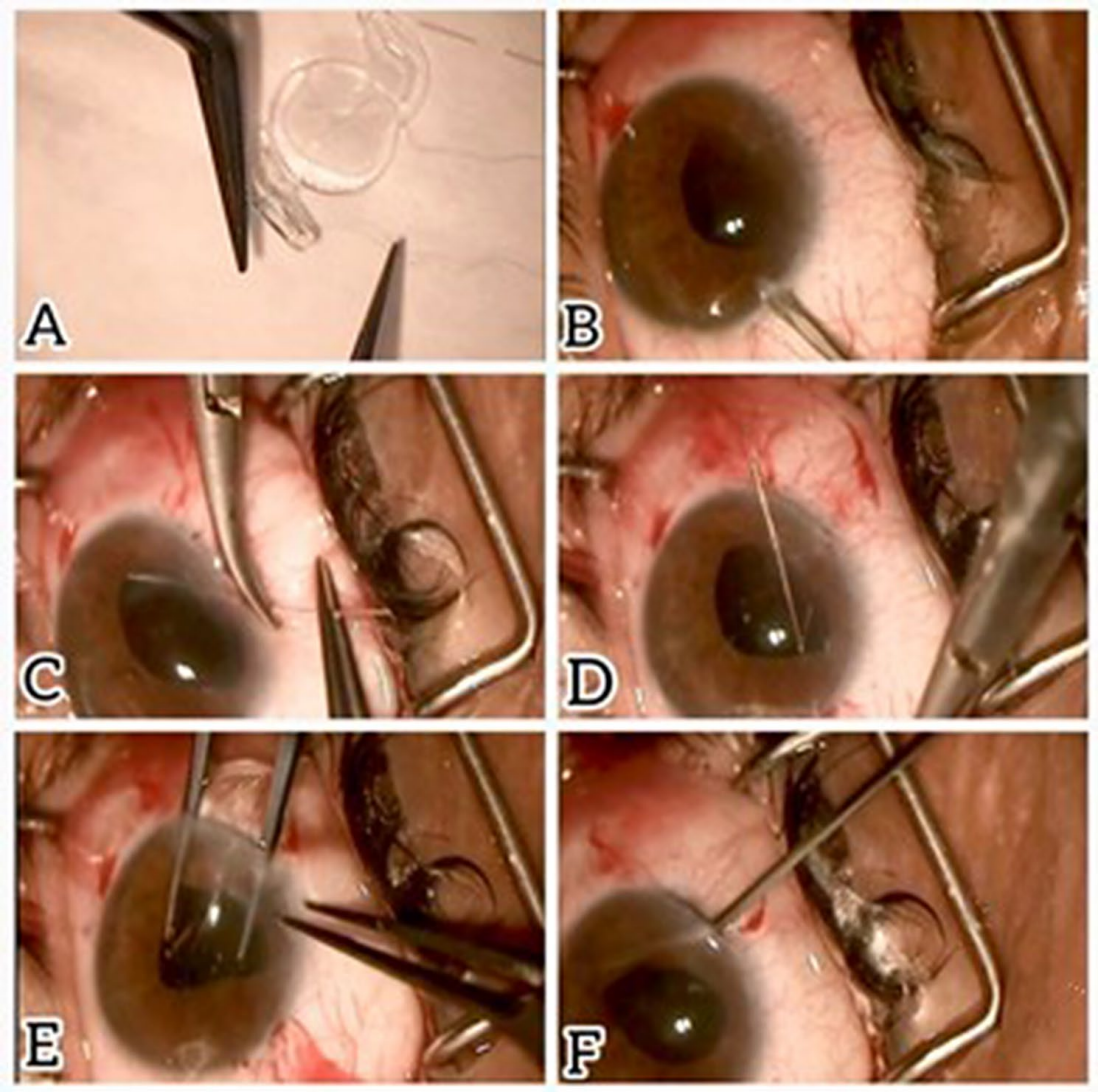
Additional procedural details for iris-suture fixation. **(A)** Haptic secured with a 10 − 0 polypropylene suture loop. **(B)** A 3.5-mm clear corneal incision and two paracenteses are created. **(C–D)** The suture needle pierces the iris periphery on opposite sides. **(E)** The foldable intraocular lens (IOL) is implanted into the anterior chamber. **(F)** Final IOL position secured within the ciliary sulcus via iris fixation

A single-piece hydrophilic acrylic posterior chamber IOL with a 6.0-mm optic and double-haptic design (*Sida IOL; Sidapharm*,* Thessaloniki*,* Greece*) was secured using 10 − 0 polypropylene (Prolene) sutures passed through the mid-peripheral iris to fixate each haptic. Sutures were tied with balanced tension, and knots were rotated and buried within the iris stroma to reduce the risk of erosion, chronic inflammation, or pupil distortion **(**Figs. 3 and 4**).**

### Preoperative and postoperative assessment

The primary objective was to evaluate postoperative visual performance using best-corrected visual acuity (BCVA). Secondary outcomes included measures of optical stability and safety: IOL centration and tilt, postoperative IOP behavior, and the incidence of surgery-related complications such as CME. All adverse events were systematically documented at each postoperative visit through standardized clinical examination and imaging per protocol-defined criteria.

Preoperative evaluation comprised uncorrected and best-corrected visual acuity, manifest refraction, slit-lamp biomicroscopy, gonioscopy, applanation tonometry, keratometry, specular microscopy for endothelial cell density, dilated fundus examination, and macular optical coherence tomography (OCT). Intraocular lens power was calculated preoperatively using optical biometry (IOL Master; Carl Zeiss Meditec), selecting the appropriate formula (e.g., SRK/T, Haigis, or Barrett Universal II) based on axial length and anterior segment parameters, with a target of emmetropia.

All postoperative measurements were performed by masked investigators. Postoperative assessments at each scheduled visit included: BCVA and refractive outcomes; IOP measurement; IOL centration and tilt evaluation using Scheimpflug imaging (Pentacam; IOL tilt was defined as the angular deviation of the optic from the pupillary plane on Scheimpflug imaging); Corneal endothelial cell density; Macular OCT for CME; Documentation of intraoperative and postoperative complications.

### Statistical analysis

Sample size was calculated using G*Power software (v3.1.9.4) [18], assuming a two-tailed α error of 0.05, power of 95%, and an effect size of 0.95. Based on CDVA data from a previous study [19] (mean 0.22 ± 0.116 in the iris-claw group vs. 0.10 ± 0.032 in the scleral fixation group; *P* = 0.00021), a total of 50 eyes (25 per group) was determined to be sufficient.

Statistical analyses were performed using Jamovi (v2.3; The Jamovi Project, Sydney, Australia). Continuous variables (age, surgical duration, IOP, CDVA in logMAR, spherical equivalent (SE), and endothelial cell count) were expressed as mean ± standard deviation with ranges. Categorical variables (sex, laterality, etiology of Aphakia, and complication rates) were expressed as frequencies and percentages. Between-group comparisons of continuous variables employed the independent samples *t*-test, while categorical variables were analyzed using the chi-squared test. *P* < 0.05 was considered statistically significant.

## Results

### Demographic data

Table 1 showed that there were no statistically significant differences between the two groups regarding age (*p* = 0.234), sex distribution (*p* = 0.258), ocular laterality (*p* = 1.000), underlying causes of Aphakia (*p* = 0.812), and mean number of previous intraocular surgeries (*p* = 0.163).

**Table 1:** Baseline demographic and clinical characteristics of the studied groups

Variable	Iris Claw IOL (*N* = 25)	Iris Suture Fixation (*N* = 25)	Total (*N* = 50)	*p* value
Age (years)				
Mean ± SD	69.0 ± 5.8	71.0 ± 5.7	70.0 ± 5.8	0.234
Range	59–80	61–81	59–81	
Sex				
Female, n (%)	10 (40.0%)	14 (56.0%)	24 (48.0%)	0.258
Male, n (%)	15 (60.0%)	11 (44.0%)	26 (52.0%)	
Eye laterality				
Left eye, n (%)	12 (48.0%)	12 (48.0%)	24 (48.0%)	1.000
Right eye, n (%)	13 (52.0%)	13 (52.0%)	26 (52.0%)	
Etiology of Aphakia				
Complicated cataract surgery	7 (28.0%)	5 (20.0%)	12 (24.0%)	0.812
Dislocated IOL	5 (20.0%)	5 (20.0%)	10 (20.0%)	
Pseudoexfoliation	7 (28.0%)	10 (40.0%)	17 (34.0%)	
Trauma	6 (24.0%)	5 (20.0%)	11 (22.0%)	
Previous surgeries				
Mean ± SD	1.8 ± 0.8	2.1 ± 0.8	2.0 ± 0.8	0.163
Range	1–3	1–4	1–4	

SD: standard deviation; IOL: intraocular lens

### Preoperative and intraoperative data

Preoperative parameters were well-matched between groups **(**Table 2**)**. Mean surgical duration was significantly shorter in the iris-claw group (30.5 ± 3.2 min) compared with the iris-suture group (46.4 ± 4.5 min; *p* < 0.001). No significant differences were observed in implanted IOL power (*p* = 0.092), preoperative IOP (*p* = 0.115), CDVA (*p* = 0.350), or corneal endothelial cell count (*p* = 0.178). Anterior vitrectomy was performed in cases with vitreous prolapse into the anterior chamber. It was required more frequently in the iris-suture group (20.0%) than in the iris-claw group (8.0%), reflecting the longer and more complex manipulation needed for suture fixation. Mean duration of anterior vitrectomy was slightly longer in the iris-suture group (3.8 ± 1.2 min) versus the iris-claw group (2.1 ± 0.9 min; *p* = 0.091).

**Table 2:** Preoperative and intraoperative characteristics of the studied groups

Variable	Iris Claw IOL (*N* = 25)	Iris Suture Fixation (*N* = 25)	Total (*N* = 50)	*p* value
Surgical time (min)				< 0.001
Mean ± SD	30.5 ± 3.2	46.4 ± 4.5	38.5 ± 8.9	
Range	25.0–36.0	39.0–55.0	25.0–55.0	
IOL power (D)				0.092
Mean ± SD	21.5 ± 1.1	22.1 ± 1.4	21.8 ± 1.3	
Range	18.8–23.5	19.0–24.2	18.8–24.2	
Preoperative IOP (mmHg)				0.115
Mean ± SD	16.6 ± 1.8	17.4 ± 2.1	17.0 ± 2.0	
Range	14.0–20.0	14.0–22.0	14.0–22.0	
Preoperative CDVA (logMAR)				0.350
Mean ± SD	1.1 ± 0.1	1.2 ± 0.2	1.1 ± 0.2	
Range	0.9–1.4	0.8–1.4	0.8–1.4	
Preoperative endothelial cell count (cells/mm²)				0.178
Mean ± SD	2459.6 ± 283.7	2337.6 ± 344.3	2398.6 ± 318.2	
Range	1980–2850	1850–2950	1850–2950	

IOL: intraocular lens; IOP: intraocular pressure; CDVA: corrected distance visual acuity; logMAR: logarithm of the minimum angle of resolution; SD: standard deviation. *p* < 0.001: highly statistically significant

### Visual and refractive results

Visual recovery was more rapid in the iris-claw group **(**Table 3**)**. At 1 month, mean CDVA was significantly better in the iris-claw group than in the iris-suture group (0.2 ± 0.1 vs. 0.3 ± 0.1 logMAR; *p* = 0.026). This difference persisted at 3 months (0.1 ± 0.1 vs. 0.2 ± 0.1 logMAR; *p* = 0.046) but diminished thereafter, with no significant differences at 6 months (*p* = 0.210) or 12 months (*p* = 0.230). At final follow-up, all eyes in both groups achieved CDVA of 20/40 or better (100%; *p* = 1.000).

Refractive outcomes approached emmetropia in both groups (Table 4). Mean SE at 1 month was − 0.3 ± 0.4 D in the iris-claw group and − 0.5 ± 0.5 D in the iris-suture group (*p* = 0.080). Similar non-significant trends favoring slightly less myopic refraction in the iris-claw group were observed at 3, 6, and 12 months (all *p* ≈ 0.076). Residual astigmatism was slightly lower in the iris-claw group (− 0.85 ± 0.61 D vs. −1.21 ± 0.74 D; *p* = 0.04). This is likely due to the sutureless fixation of the iris-claw IOL itself, which allows symmetric enclavation and minimizes rotational instability. Although two 10 − 0 nylon sutures were used to close the corneal incision, they do not affect IOL orientation. In contrast, iris-sutured IOLs may experience slight haptic torque or tilt during knot tying, contributing to higher residual astigmatism.

**Table 3:** Postoperative visual outcomes in the studied groups

Timepoint	Iris Claw IOL (*N* = 25)	Iris Suture Fixation (*N* = 25)	Total (*N* = 50)	*p* value
CDVA (logMAR) 1 month	0.2 ± 0.1	0.3 ± 0.1	0.2 ± 0.1	0.026*
Range	0.0–0.4	0.0–0.5	0.0–0.5	
CDVA (logMAR) 3 months	0.1 ± 0.1	0.2 ± 0.1	0.2 ± 0.1	0.046*
Range	0.0–0.3	-0.1–0.4	-0.1–0.4	
CDVA (logMAR) 6 months	0.1 ± 0.1	0.2 ± 0.1	0.1 ± 0.1	0.210
Range	0.0–0.3	0.0–0.3	0.0–0.3	
CDVA (logMAR) 12 months	0.1 ± 0.1	0.1 ± 0.1	0.1 ± 0.1	0.230
Range	-0.1–0.3	-0.1–0.3	-0.1–0.3	
Final CDVA ≥ 20/40, n (%)	25 (100%)	25 (100%)	50 (100%)	1.000

CDVA: corrected distance visual acuity; logMAR: logarithm of the minimum angle of resolution; SD: standard deviation; *p < 0.05, statistically significant

**Table 4:** Postoperative refractive outcomes (spherical equivalent)

Timepoint	Iris Claw IOL (*N* = 25)	Iris Suture Fixation (*N* = 25)	Total (*N* = 50)	*p* value
SE (D) 1 month	−0.3 ± 0.4	−0.5 ± 0.5	−0.4 ± 0.5	0.080
Range	−1.0–0.4	−1.3–0.6	−1.3–0.6	
SE (D) 3 months	−0.3 ± 0.4	−0.5 ± 0.5	−0.4 ± 0.5	0.076
Range	−1.0–0.4	−1.2–0.5	−1.2–0.5	
SE (D) 6 months	−0.3 ± 0.4	−0.5 ± 0.5	−0.4 ± 0.5	0.076
Range	−1.0–0.4	−1.2–0.5	−1.2–0.5	
SE (D) 12 months	−0.3 ± 0.4	−0.5 ± 0.5	−0.4 ± 0.5	0.076
Range	−1.0–0.4	−1.2–0.5	−1.2–0.5	

SE: spherical equivalent; D: diopter; SD: standard deviation

### Postoperative endothelial cell count

Endothelial cell loss at 12 months was significantly greater in the iris-claw group (14.5% ± 4.2) compared with the iris-suture group (9.8% ± 3.5; *p* < 0.001). The difference reflects closer anterior chamber manipulation and transient corneal contact during enclavation.

### Postoperative IOP

**Fig. 5:**
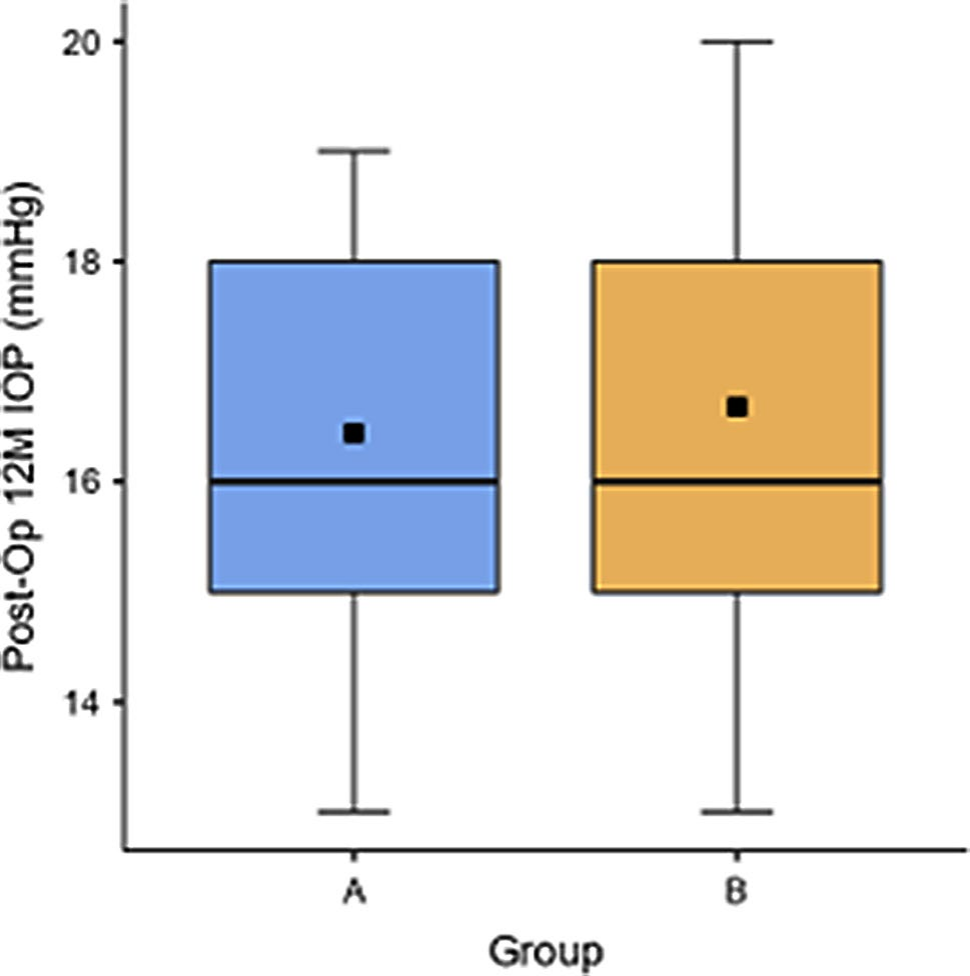
Mean (± standard deviation) intraocular pressure (IOP) at 12 months postoperatively in the iris-claw and iris-suture fixation groups

Both groups exhibited transient IOP elevation in the early postoperative period, with values gradually returning toward baseline by 12 months (Fig. 5). In the iris-claw group, mean IOP increased from 16.6 ± 1.8 mmHg preoperatively to 18.9 ± 3.0 mmHg at 1 week, then declined to 16.8 ± 2.4 mmHg at 12 months. Corresponding values in the iris-suture group were 17.4 ± 2.1 mmHg preoperatively, 19.2 ± 3.3 mmHg at 1 week, and 17.1 ± 2.6 mmHg at 12 months. No statistically significant intergroup differences were observed at any time point (*p* > 0.05).

### Intraoperative and postoperative complications

The overall incidence of complications was similar between groups (44.0% in the iris-claw group vs. 52.0% in the iris-suture group; *p* = 0.777), with one notable exception: corneal endothelial cell loss was significantly greater in the iris-claw group (14.5% ± 4.2 vs. 9.8% ± 3.5; *p* < 0.001).

Regarding intraoperative complications, transient hyphema occurred in 4.0% vs. 12.0% of eyes (*p* = 0.609), and iris tears or iridodialysis in 4.0% vs. 8.0% (*p* = 1.000). Anterior vitrectomy was performed more frequently in the iris-suture group (20.0% vs. 8.0%; *p* = 0.184), with a longer mean duration (3.8 ± 1.2 min vs. 2.1 ± 0.9 min; *p* = 0.091). This higher frequency likely reflects the increased manipulation required for haptic suturing, which can increase the risk of vitreous prolapse.

**Fig. 6:**
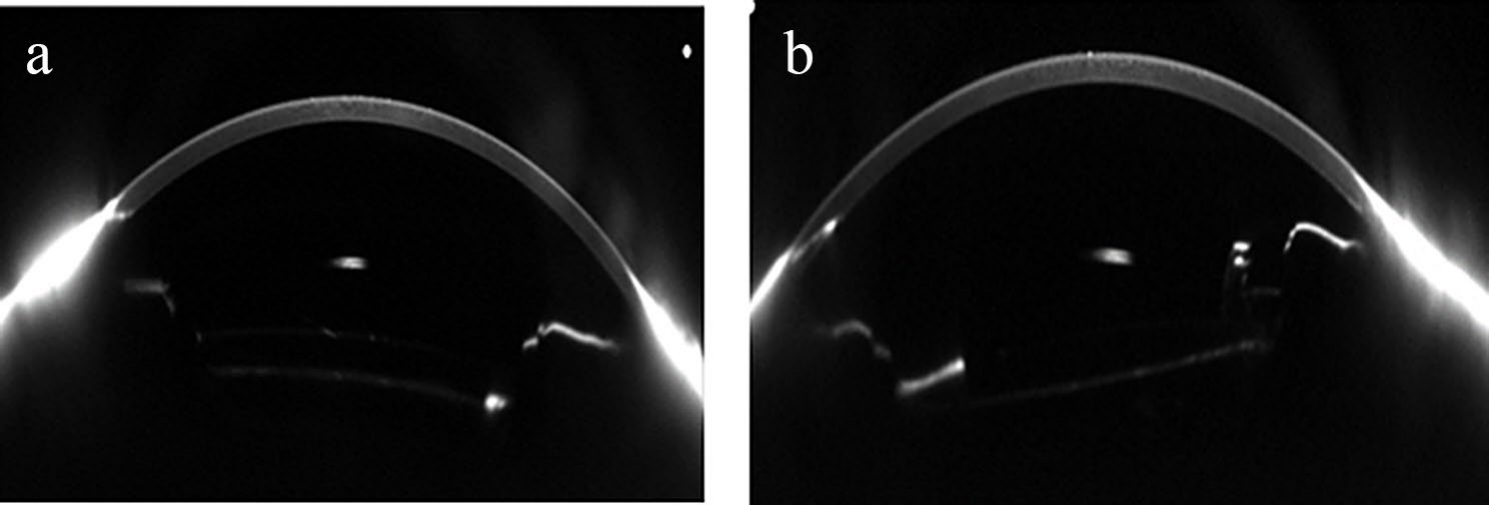
Scheimpflug imaging (Oculus Pentacam) of intraocular lens (IOL) position in the iris-claw group. **(A)** Well-centered IOL with no detectable decentration or tilt. **(B)** Tilted IOL showing asymmetric haptic positioning relative to the pupillary plane, illustrating variability in postoperative IOL alignment

**Fig. 7:**
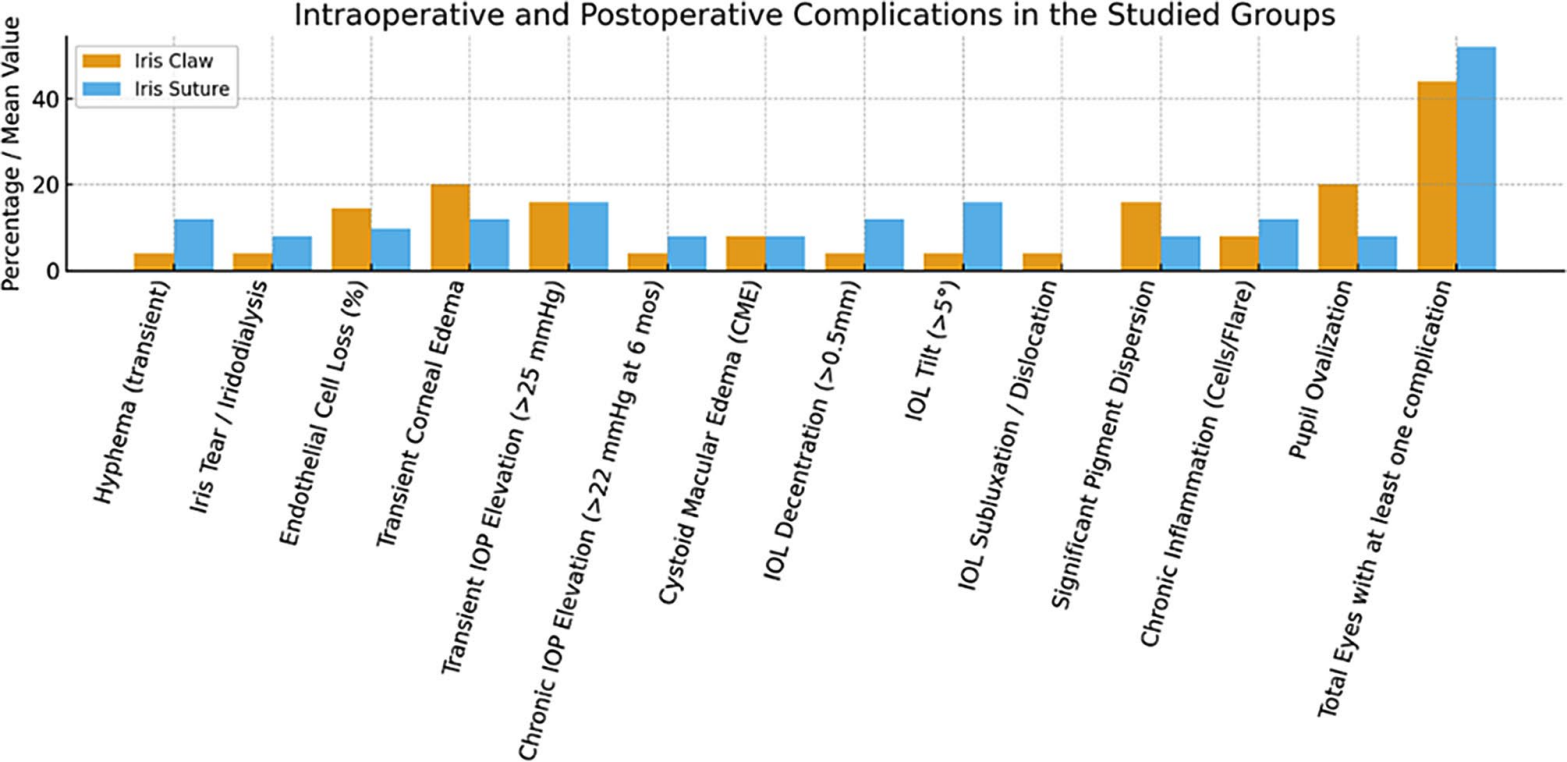
Frequency of intraoperative and postoperative complications by treatment group. Orange bars represent the iris-claw group; blue bars represent the iris-suture fixation group. Complications assessed include transient hyphema, iris tear or iridodialysis, percentage of endothelial cell loss, transient corneal edema, transient intraocular pressure elevation (> 25 mmHg), chronic intraocular pressure elevation (> 22 mmHg at 6 months), cystoid macular edema, intraocular lens (IOL) decentration (> 0.5 mm), IOL tilt (> 5°), IOL subluxation or dislocation, significant pigment dispersion, chronic inflammation (cells/flare), and pupil ovalization. The proportion of eyes experiencing at least one complication is also shown

Concerning postoperative complications, transient corneal edema was observed in 20.0% vs. 12.0% of eyes (*p* = 0.702). Transient and chronic IOP elevations were comparable between groups (*p* = 1.000). CME developed in 8.0% of eyes in each group. IOL-related events, including decentration (> 0.5 mm; 4.0% vs. 12.0%), tilt (> 5°; 4.0% vs. 16.0%), and subluxation/dislocation (4.0% vs. 0%), did not differ significantly (all *p* > 0.05). Iris-related complications (pigment dispersion, chronic inflammation, pupil ovalization) were similarly distributed. Representative imaging findings are shown in Figs. 6 and 7.

## Discussion

This prospective, randomized controlled trial compared the functional and safety outcomes of retropupillary iris-claw fixation and iris-suture fixation for posterior chamber IOL implantation in aphakic eyes lacking adequate capsular support. Both techniques yielded substantial improvements in CDVA relative to baseline, confirming their efficacy for visual rehabilitation in this challenging clinical scenario.

Early visual recovery was more rapid in the iris-claw group, with significantly better CDVA at 1 and 3 months. By 6 and 12 months, visual outcomes converged, demonstrating equivalent long-term efficacy. These findings suggest that retropupillary iris-claw fixation facilitates faster initial visual rehabilitation without compromising final visual acuity. This pattern aligns with prior reports: Drolsum and Kristianslund [20] documented rapid visual improvement with iris-claw IOLs and comparable long-term outcomes, whereas Madhivanan et al. [21] observed no significant differences in final CDVA between iris-claw and iris-suture fixation techniques.

Intraoperative complications were infrequent and similarly distributed between groups. Transient hyphema and minor iris trauma occurred at low rates, while anterior vitrectomy was required more frequently in the iris-suture group, reflecting greater vitreous disturbance during suture manipulation [22, 23]. These observations support the overall safety of both approaches when performed by experienced anterior segment surgeons, though iris-suture fixation entails greater technical complexity.

A key safety distinction emerged in corneal endothelial cell loss, which was significantly greater in the iris-claw group. This finding is consistent with literature reporting increased endothelial impact with iris-claw IOLs [24], despite the retropupillary position minimizing direct endothelial contact [25]. Other postoperative complications, including transient corneal edema, IOP fluctuations, CME, and iris-related events, occurred at low and comparable frequencies. Given the overall low incidence of adverse events, assertions of equivalent safety profiles should be interpreted cautiously, as the study may have been underpowered to detect differences in rare complications.

IOL stability parameters, assessed objectively using Scheimpflug imaging, revealed a trend toward greater decentration and tilt in the iris-suture group, although differences did not reach statistical significance. These observations accord with previous reports of late IOL displacement and suture-related complications in iris-sutured lenses [26]. Retropupillary iris-claw IOLs generally maintained stable fixation, with disenclavation occurring infrequently [27].

Postoperative IOP profiles were comparable between groups, characterized by transient early elevation followed by normalization over 12 months. A marginally higher frequency of chronic IOP elevation was noted in the iris-suture group, potentially attributable to suture-induced inflammation or pigment dispersion [28–30]. The absence of prolonged IOP elevation in the iris-claw group supports its suitability in eyes without preexisting glaucomatous damage [20, 21].

CME developed in 8.0% of eyes in each group, consistent with previously reported rates of 5% to 10% following secondary IOL implantation [31, 32]. OCT confirmed comparable central macular thickness at 12 months, indicating similar retinal safety profiles [33, 34].

In summary, both retropupillary iris-claw fixation and iris-suture fixation represent effective strategies for visual rehabilitation in aphakic eyes without capsular support. Iris-claw fixation offers advantages of shorter operative duration (30.5 vs. 46.4 min), accelerated early visual recovery, and mechanically stable sutureless fixation. However, it is associated with greater endothelial cell loss, warranting caution in eyes with compromised endothelial reserves. Iris-suture fixation provides a viable alternative in such cases, avoiding direct endothelial contact and permitting foldable IOL implantation through smaller incisions, albeit with increased surgical complexity and potential for suture-related complications.

This study has several limitations. The sample size, while adequate for the primary outcome, may have limited power to detect differences in rare complications. The 12-month follow-up period is insufficient to evaluate late events such as suture degradation, delayed disenclavation, or progressive endothelial attrition or iris atrophy and long-term changes at the site of Artisan/iris-claw lens enclavation. Furthermore, procedures were performed at tertiary referral centers by highly experienced surgeons, which may limit generalizability to broader clinical settings. Moreover, including patient-reported outcomes would provide valuable insight into the functional and quality-of-life impact of each technique. Future investigations should incorporate larger, multicenter cohorts with extended follow-up durations, alongside direct comparisons of anterior versus retropupillary iris-claw fixation and emerging sutureless scleral fixation techniques.

### Clinical implications

Both techniques permit individualized surgical planning based on ocular characteristics and clinical priorities. Retropupillary iris-claw fixation may be preferred in eyes with healthy corneal endothelium when rapid visual rehabilitation and reduced operative time are prioritized. Conversely, iris-suture fixation represents a suitable alternative in eyes with marginal endothelial reserves, as it avoids direct endothelial contact and accommodates foldable IOLs through smaller incisions. Regardless of technique selection, optimal outcomes depend on advanced surgical expertise to ensure precise IOL positioning and minimize procedure-related complications.

## Conclusion

Retropupillary iris-claw fixation and iris-suture fixation both provide effective visual rehabilitation for aphakic eyes without adequate capsular support, achieving comparable long-term visual acuity with 100% of eyes attaining 20/40 or better vision at 12 months. Iris-claw fixation offers the advantages of shorter surgical duration and faster early visual recovery, whereas iris-suture fixation is associated with less endothelial cell loss. Both techniques demonstrate acceptable safety profiles, with low rates of sight-threatening complications. The choice between approaches should be individualized based on preoperative endothelial status, surgical expertise, and patient-specific factors influencing the relative importance of rapid visual rehabilitation versus endothelial preservation.

## Supplementary Information

Below is the link to the electronic supplementary material.

Supplementary Material 1

## Data Availability

The datasets generated and/or analyzed during the current study are available from the corresponding author on reasonable request.

## Abbreviations

BCVA: Best-Corrected Visual Acuity
CDVA: Corrected Distance Visual Acuity
CME: Cystoid Macular Edema
IOL: Intraocular Lens
IOP: Intraocular Pressure
logMAR: Logarithm Of The Minimum Angle Of Resolution
OCT: Optical Coherence Tomography
SD: Standard Deviation
SE: Spherical Equivalent
